# Performance characterization of freeform finished surfaces of potassium dihydrogen phosphate using fluid jet polishing with a nonaqueous slurry

**DOI:** 10.1038/s41598-023-33695-x

**Published:** 2023-04-21

**Authors:** Nathaniel D. Urban, Kyle R. P. Kafka, Ji-Mi Jang, Brittany N. Hoffman, Kenneth L. Marshall, Rhys Emms, David Walker, Stavros G. Demos

**Affiliations:** 1 Laboratory for Laser Energetics, University of Rochester, 250 East River Road, Rochester, NY 14623-1299 USA; 2 LightMachinery Inc., 80 Colonnade Rd #1, Nepean, ON K2E 7L2 Canada

**Keywords:** Materials science, Materials for optics, Applied optics, Optical materials and structures

## Abstract

Potassium dihydrogen phosphate (KDP) and its deuterated analog (DKDP) are unique nonlinear optical materials for high power laser systems. They are used widely for frequency conversion and polarization control by virtue of the ability to grow optical-quality crystals at apertures suitable for fusion-class laser systems. Existing methods for freeform figuring of KDP/DKDP optics do not produce surfaces with sufficient laser-induced–damage thresholds (LIDT’s) for operation in the ultraviolet portion of high-peak-power laser systems. In this work, we investigate fluid jet polishing (FJP) using a nonaqueous slurry as a sub-aperture finishing method for producing freeform KDP surfaces. This method was used to selectively polish surface areas to different depths on the same substrate with removals ranging from 0.16 μm to 5.13 μm. The finished surfaces demonstrated a slight increase in roughness as the removal depth increased along with a small number of fracture pits. Laser damage testing with 351 nm, 1 ns pulses demonstrated excellent surface damage thresholds, with the highest values in areas devoid of fracture pits. This work demonstrates, for the first time, a method that enables fabrication of a waveplate that provides tailored polarization randomization that can be scaled to meter-sized optics. Furthermore, this method is based on FJP technology that incorporates a nonaqueous slurry specially designed for use with KDP. This novel nonaqueous FJP process can be also used for figuring other types of materials that exhibit similar challenging inherent properties such as softness, brittleness, water-solubility, and temperature sensitivity.

## Introduction

Both potassium dihydrogen phosphate (KDP) and its deuterated analog DKDP are critical optical materials in large-aperture, high-peak-power laser systems, where they are used for frequency conversion, polarization smoothing, and electro-optical switching^1^. Owing to their large nonlinear optical coefficient, high laser-induced–damage threshold (LIDT), and significant optical activity^2,3^, KDP and DKDP are indispensable materials for fusion-class laser systems such as the National Ignition Facility (NIF, USA) and the Laboratory for Laser Energetics’ Omega Laser Facility (LLE, USA)^4^. However, fabrication of optical quality KDP surfaces is exceedingly difficult due to the material’s high water solubility, brittleness, softness, and temperature sensitivity^5^. Because of these properties, commercial finishing of KDP and DKDP is limited to single-point diamond turning and conventional lap polishing of flats using nonaqueous cooling fluids and slurries^6,7^. In recent years, research has been conducted on various sub-aperture finishing methods for KDP. A stable, high-accuracy sub-aperture removal process would allow for freeform surface figuring of this material. In turn, this would enable fabrication of new optical elements such as wave plates with spatially variable retardance for polarization mixing (smoothing) of linearly polarized beams as well as wavefront correction of optical flats. The nature of the sub-aperture methods studied vary considerably and include magnetorheological finishing (MRF), sub-aperture polishing, ion beam figuring (IBF), ultraprecision grinding, and abrasive-free jet polishing^8–18^. Although promising, these methods can produce a variety of defects such as crack formations from heating (IBF) and iron contamination (MRF) that undermine the ability of the optic to withstand high laser power in the ultraviolet regime^8,9,13,15^. Abrasive-free jet polishing employs the use of water-in-oil microemulsions in a fluid jet to achieve removal by a dissolution mechanism^17,18^. Although the method is sub-aperture, our experience is that material removal and crystal etching can occur rapidly during contact with a microemulsion. Subsequently, surface areas far from the primary impact region of the fluid jet, yet inadvertently exposed to the microemulsion, are negatively affected. Thus, the ability of this method to produce high-quality freeform surfaces is severely restricted.

In the most basic sense, fluid jet polishing (FJP)^19^ consists of pumping a polishing slurry containing abrasive particles at low pressures (0.5 to 8 bar) through a nozzle with an outlet diameter on the order of 1 mm. The abrasive slurry impinges on the surface of the workpiece, generating a millimeter-scale removal spot. Adaptation of FJP for freeform KDP finishing using a nonaqueous slurry may possess several distinct advantages. First, active cooling of the substrate by the fluid jet reduces temperature variations that can result in catastrophic damage to the crystal. Second, there are minimal limitations on the composition of the polishing slurry, which enables the use of slurry chemistries that are compatible with KDP in terms of chemical reactivity and preservation of the laser damage performance. Fluid jet polishing also possesses the ability to remove material in both brittle and ductile modes^20,21^. In the ductile mode, impinging particles will only penetrate the surface to a shallow depth and not induce subsurface damage. As such, pre-existing damage from grinding and polishing is reduced as removal by FJP progresses. Recent studies on fused silica have shown that when using an appropriate slurry and process, FJP can maintain or even significantly enhance the LIDT^22,23^.

This article reports the laser damage performance of KDP surfaces finished by FJP to different depths using a specially developed oil-based abrasive slurry consisting of mineral oil and alumina nanoparticles. Laser damage testing was performed using 351 nm, 1 ns laser pulses. The results indicate that a reasonably high LIDT can be achieved and could be further improved by eliminating a class of pit defects that are formed sporadically during the process and act to trap residual components of the polishing slurry. Surface areas free of these defects exhibit excellent laser damage behavior.

## Experimental

### Fluid jet polishing

To prepare the nonaqueous slurry, 5 wt% sodium stearate (Sigma Aldrich) was mixed thoroughly with light mineral oil. Aluminum oxide (50-nm Average particle size) was then added at 5 wt% concentration. The formulation was filtered by passing it through a 63 μm sieve.

**Figure 1:**
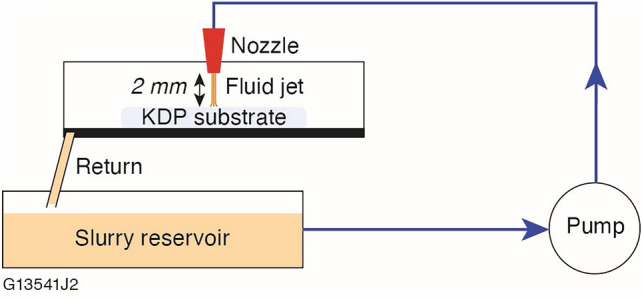
Schematic of the FJP setup used for this study.

The FJP setup used for these experiments is consistent with a basic setup as described in the literature^19,24^. A schematic of the FJP setup used for this study is shown in Fig. 1.

The nonaqueous slurry was delivered and recirculated by a diaphragm pump at 6.9 bar pumping pressure. The nozzle possessed a cylindrical, 1 mm diameter outlet and was oriented perpendicular to the mounted substrate at a 2 mm standoff distance. The process is computer numerically controlled (CNC) as the nozzle can raster over the surface of the workpiece. The CNC software inputs a tool spot removal profile and determines the path and dwell times necessary to achieve the desired surface profile. Stability of both the process and slurry was monitored by periodically evaluating the tool spot removal profile for any changes. Post-FJP cleaning was performed by submerging the sample in sequential baths of isopropanol followed by rinsing with xylenes and gently drag wiping the sample with a non-woven clean room wiper. The KDP crystals used for this study were 1.0 cm thick, 2.5 and 5.0 cm square substrates hand polished on a pitch lap. The optic axis was 90° (X-cut) with respect to normal on the surface of the sample.

### Surface analysis

Surface profile measurements and rms roughness analysis were conducted on a Zygo Nexview, white-light interferometer. Roughness analysis was measured over an 800 × 800 μm area using a 2.8 × objective. The Nomarski differential interference contrast (DIC) micrographs were captured using a Zeiss Axio microscope operating in reflection mode with a 100 × objective. Scanning electron microscopy/energy dispersive x-ray spectroscopy (SEM/EDS) analysis was conducted on a Zeiss Sigma 300 (SEM) and EDAX Octane Elect (EDS). Prior to analysis, the KDP substrate was sputter coated with platinum.

### Laser source and damage testing protocols

Laser damage experiments were conducted using a *Q*-switched, Nd-YLF laser, frequency tripled to 351 nm with a 1 ns pulse duration. The experimental format is consistent with a standardized approach to laser damage testing^25^. The Gaussian beam (350 μm, 1/*e*^2^) was focused at a 7° angle of incidence on the front surface of the KDP substrate. Laser-induced damage was monitored using an *in-situ* dark-field microscope with white-light illumination. Detection of damage was determined using subtraction between images of the site taken before and after irradiation.

The testing protocol for fracture pits consisted of a multi-pulse fluence ramp (*R*-on-1) using a 0.1 Hz repetition rate and initial fluence of 3 J/cm^2^. The fluence was then ramped in 5% relative increments until damage was observed. The seven largest fracture pits were laser damage tested in total.

For surfaces devoid of fracture pits, a 1-on-1 testing protocol was employed. This protocol exposes each new site to a single laser pulse at a particular fluence and records the presence or absence of damage on the surface in a binary manner. For each FJP-finished surface, ~ 100 testing sites were used. The damage probabilities were calculated using the “limit cumulative method”^26^ and the LIDT values correspond to a damage probability of 5% to 10%.

## Results and discussion

### Fluid jet polishing

The nonaqueous slurry used for this study was developed with the goal of achieving both compatibility with KDP and maximum LIDT. Light mineral oil was found to be a suitable carrier liquid due to both its intrinsically low water content (which was found to be important for minimizing process-induced defects) and inability to absorb atmospheric moisture. Additionally, due to its low vapor pressure (< 0.1 mmHg at 20 °C), negligible evaporation occurs over the course of long polishing runs, which could result in modified removal characteristics. Based on previous testing using aqueous slurries for fused silica polishing^22^, alumina particles possess a low absorption at 351 nm, which reduces the impact on LIDT from particle contamination on the surface as compared to other abrasives such as ceria. Sodium stearate functions as a dispersant to keep the alumina particles suspended and deagglomerated by means of steric repulsion. Rapid settling of the particles can result in inconsistencies of the removal rate. The addition of viscosity modifiers can also have effects on slurry performance, since these additives can change parameters related to particle movements and distribution within a fluid jet^24^.

**Figure 2:**
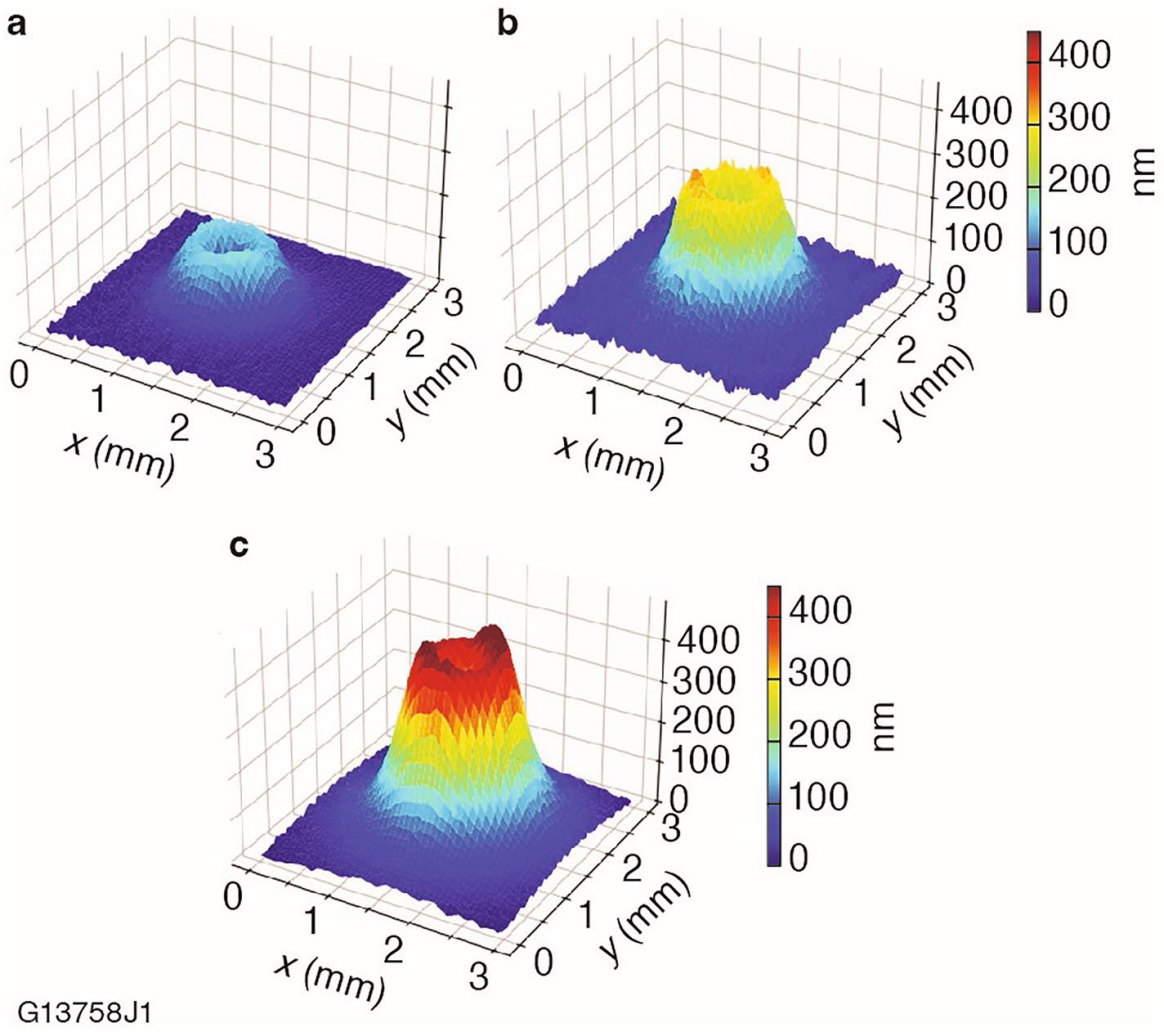
Fluid jet polishing tool spot profile on KDP substrates generated by various process changes including pressure, nozzle diameter, standoff distance, and formulation modifications.

The FJP process was developed primarily to achieve a high removal rate that would allow for processing large-aperture optics. Secondary considerations of importance were surface roughness and the full width at half maximum (FWHM) tool spot size. Although there are numerous process parameters that can be tuned for an FJP process, the primary considerations for this work included abrasive content, nozzle diameter, standoff distance, and slurry delivery pressure. Three tool spot profiles generated by 30 s dwell times from this optimization process are shown in Fig. 2.

The FJP process that demonstrated optimal performance based on achieving an application-relevant removal rate with acceptable roughness and FWHM, generated the tool spot in Fig. 2c. The parameters for this process are outlined in the Experimental section above. The removal rate achieved with this process was 5.9 μm^3^/s with a FWHM value of 1.4 mm. For our intended application, a 1.4 mm FWHM enables us to fabricate features suitable for randomizing the polarization of a 300 mm diameter laser beam by introducing a predetermined spatial pattern of the retardance of the KDP wave plate. Faster removal rates are achievable with nozzle diameters larger than 1 mm (using the same parameters), but the increase in FWHM lowers the spatial resolution that can be achieved.

### Test sample fabrication

**Figure 3:**
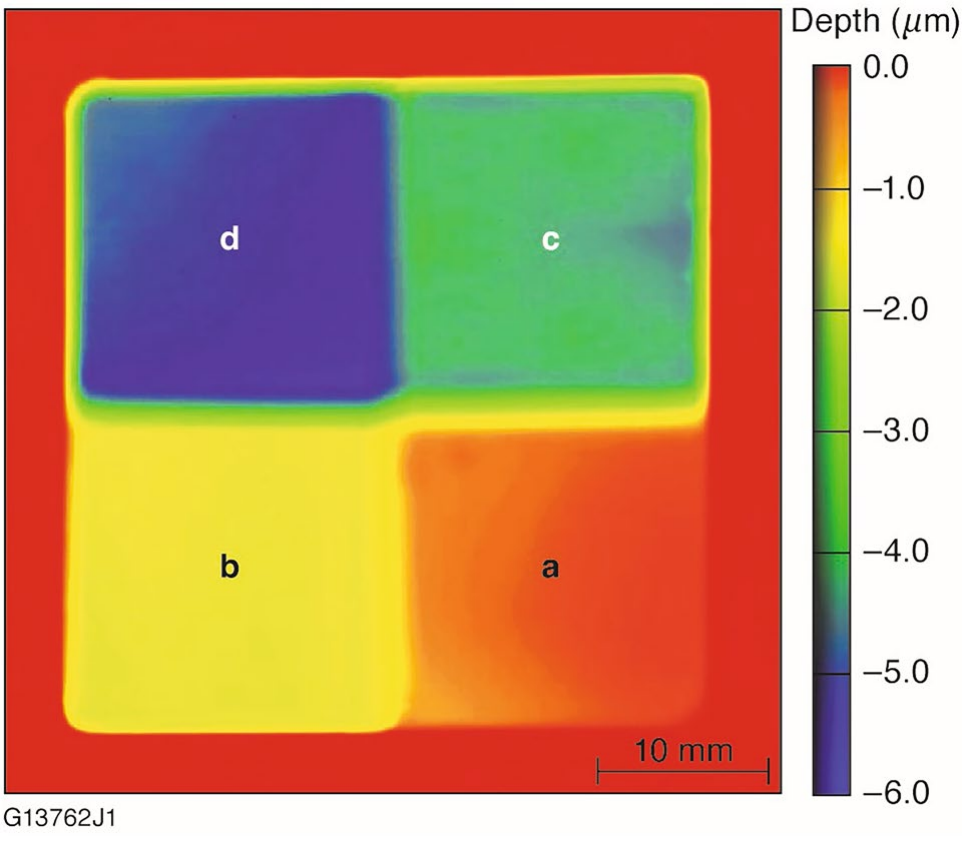
Surface profile of the X-cut KDP substrate fabricated for this study: (**a**) 0.16 μm, (**b**) 1.59 μm, (**c**) 4.19 μm, and (**d**) 5.13 μm.

The laser damage testing and characterization results presented in this work were obtained from four different surface depths generated by the FJP process discussed above. Since the LIDT can vary between KDP substrates for various reasons that are outside of our control^27,28^, the surface depths were all generated on a single substrate. The finished surfaces were 1.5 × 1.5 cm square areas with FJP removal depths of 0.16 μm, 1.59 μm, 4.19 μm, and 5.13 μm. A maximum removal depth of 5.13 μm was chosen because it represents the largest surface height variation required for our polarization-smoothing application of interest. The KDP crystal surface (5.0 × 5.0 × 1.0 cm) used for this study was in an X-cut (90° from optic axis) orientation and polished on a pitch lap. During fabrication, the tool spot profile generated by the slurry was periodically tested and measured to ensure both process stability and that significant changes such as particle agglomeration were not occurring. No measurable change in the tool spot profile was observed upon completion of the run. For larger optics requiring more material removal, this periodic monitoring would be important to account for any drifting of the tool spot profile over a longer polishing run. A surface profile measurement of the finished, freeform test substrate is shown in Fig. 3.

### Surface characterization

Roughness measurements of the four, FJP-finished surfaces (800 × 800 μm analysis area) are shown in Table 1. This analysis reveals an increase in rms roughness as the amount of material removed by FJP progressed.

**Table 1:** Roughness measurements for the 15 × 15-mm, FJP-finished surfaces.

Depth (μm)	rms roughness (nm)
0 (as received)	0.9
0.16	2.6
1.59	5.4
4.19	6.8
5.13	8.0

An increase in roughness after FJP of a highly polished surface is typical and is indicative that material removal is occurring by both ductile and brittle fracture modes^20,21,24^. This behavior aligns with proposed FJP removal mechanisms for soft and brittle materials such as KDP^29^. The transition between these removal modes for a particular material occurs when impacting particles reach a critical penetration depth *d*_c_ as defined by Bifano^30^.

1 $$d_{{\text{c}}} = 0.15\left( \frac{E}{H} \right)\left( \frac{Kc}{H} \right)^{2} .$$

The fracture toughness of the material is defined as *k*_c_, *E* is the elastic modulus, and *H* is the hardness. Abrasive particles with enough energy to penetrate the surface of the workpiece beyond this depth will generate cracks and remove material from the substrate in the brittle mode. For the X-cut surface plane of KDP used for this study, we input *k*_c_ = 0.22 MPa · m^1/2^, *E* = 38.7 GPa, and *H* = 1.4 GPa, which produces a critical depth value of 102 nm. It is important to note that the mechanical properties of KDP vary in the literature and demonstrate anisotropic and load-dependent physical properties even within a single crystal plane^31^. However, since our tool spot profile exceeds 400 nm in depth, we assume brittle fracture is occurring as a component of the removal process.

**Figure 4:**
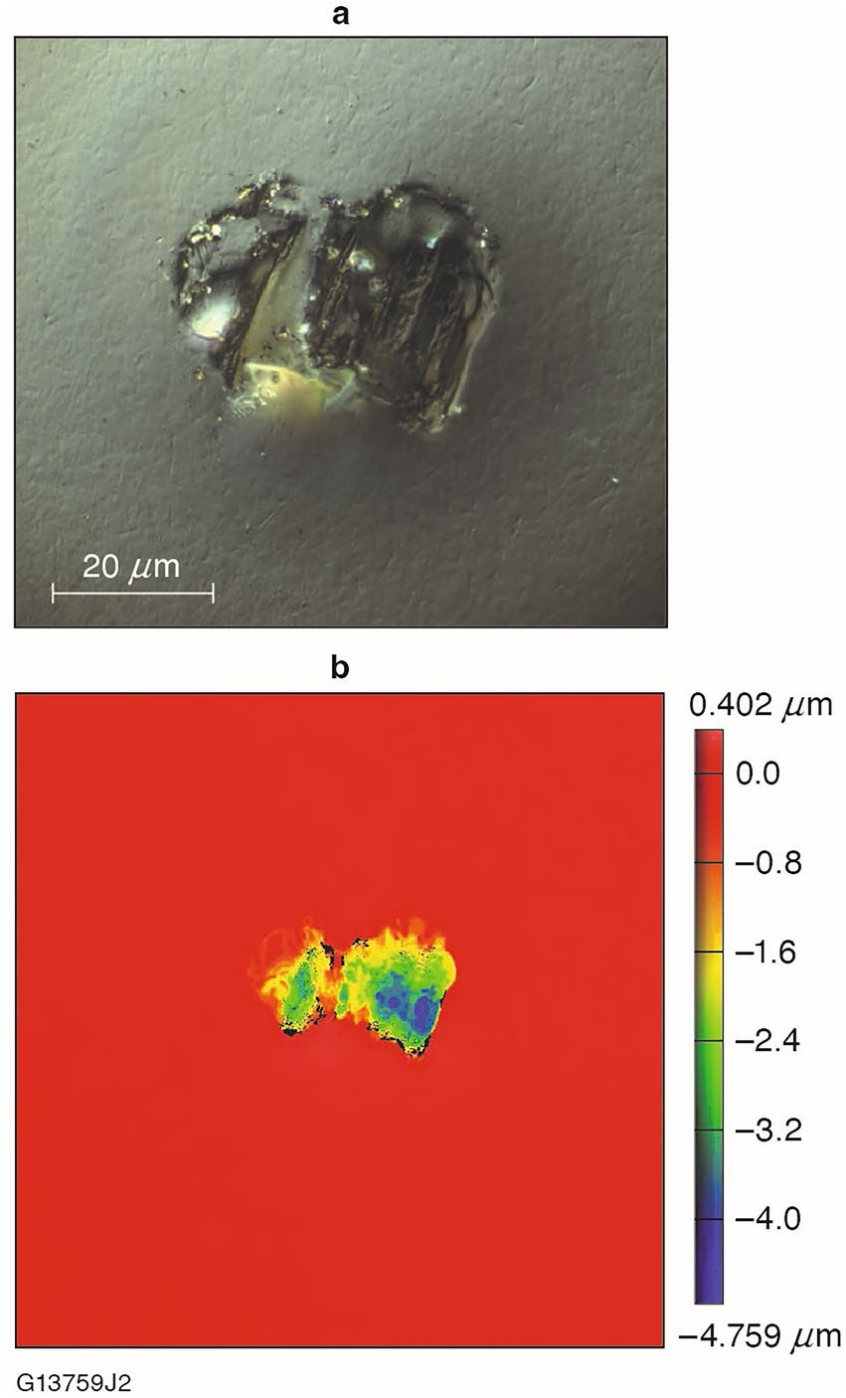
(**a**) Micrograph of a representative fracture pit observed after FJP finishing. (**b**) Surface profile of the same pit as measured on a white-light interferometer. The maximum depth of the pit is 4.6 μm.

Further analysis of the FJP-finished substrate by Nomarski DIC microscopy revealed the presence of a small number of fracture pits such as the example shown in Fig. 4.

Full quantification of the pits revealed their number density to be substantially higher in the deeper (4.19 μm and 5.13 μm) squares as exhibited in Table 2.

**Table 2:** Number density of the fracture pit defects.

Depth (μm)	Pits per cm^2^
0.16	0.6
1.59	2.0
4.19	7.3
5.13	12.0

Due to the low number and sporadic formation of the pits, our hypothesis is that their formation is driven by the presence of subsurface crystal defects. A variety of bulk defects have been previously identified in KDP such as dislocations^32,33^, liquid inclusions^34^, and even clusters that have demonstrated mobility under laser excitation^35^. These defects create areas of strain in the near-surface of KDP crystals, thus are more susceptible to mechanical failure and initiation of crack formations^36–38^. As such, the frequency of fracture pit formation is a likely indicator of crystal quality. As the depth of FJP progresses, additional defects are encountered and the probability of brittle fracture events increases. Furthermore, the depths observed for these fracture pits (3 to 7 μm), means that many of them will still exist at the deepest (5.13 μm) FJP depth investigated in this study, and their occurrence will be cumulative as depth increases. Future studies will focus on testing crystals from various growth methods and conducting FJP removal to greater depths in order to build a more comprehensive understanding of this phenomenon. Additionally, we envision that FJP may be a suitable method to access and further study these crystal defects, which are difficult to detect using existing analytical tools.

**Figure 5:**
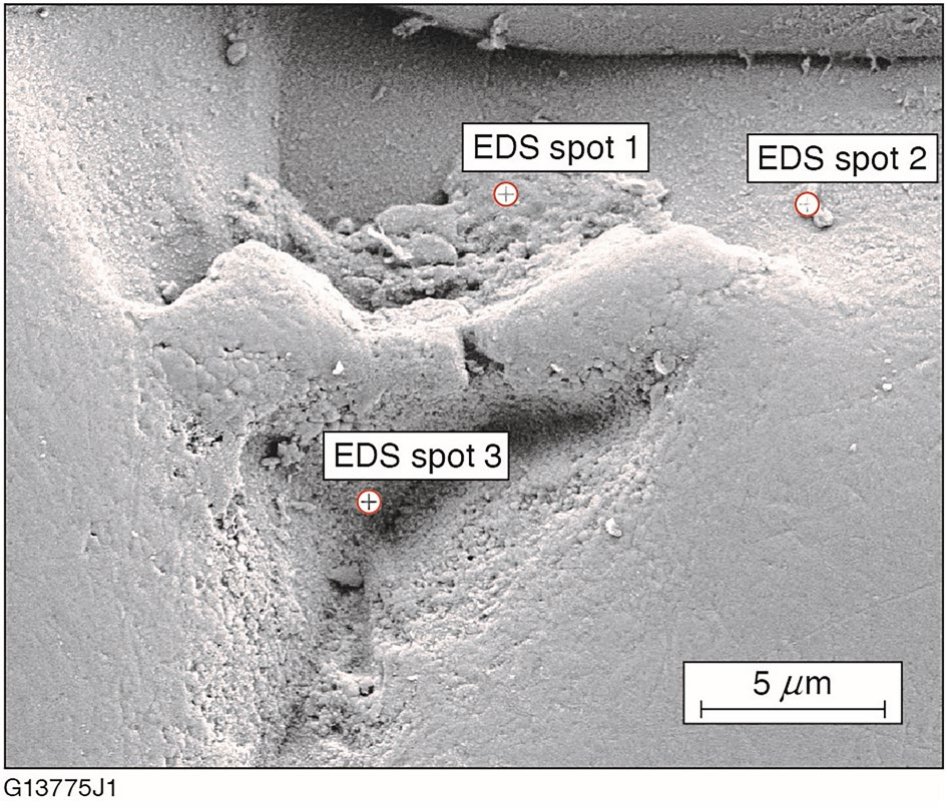
SEM image of the fracture pit and locations used to conduct EDS analysis.

Since cracks and pits are known to harbor contamination detrimental to laser damage resistance, SEM/EDS was conducted on another KDP substrate processed by FJP to a removal depth of 3.4 μm. Table 3 shows that the EDS analysis revealed the presence of residual slurry components (C, Na, Al) within one of the fracture pits. In otherwise “pristine” areas of the substrate’s surface, sodium and aluminum were undetected while the level of carbon detected was significantly diminished. An SEM image of the fracture pit and areas used for EDS analysis is shown in Fig. 5.

**Table 3:** Elemental analysis by SEM/EDS of an FJP-induced fracture pit as compared to the remainder of the surface.

	Fracture pit (wt%)	Pristine (wt%)
C	19.0	6.8
O	44.7	48.7
Na	0.4	Undetected
Al	0.4	Undetected
P	17.2	21.0
K	18.5	23.5

### Laser damage characteristics

**Figure 6:**
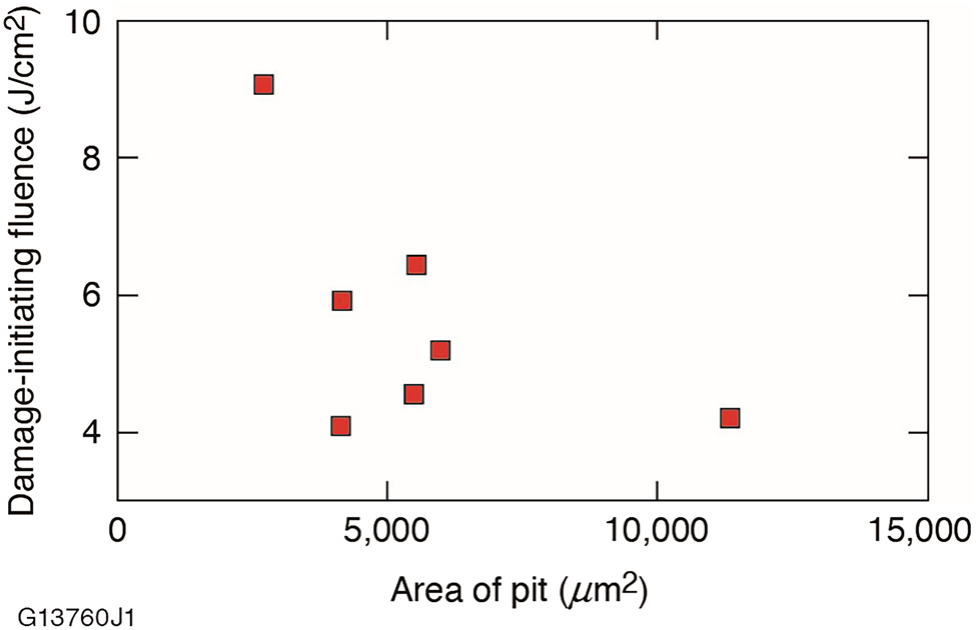
The *R*-on-1 damage-initiating fluence versus fracture pit area for the seven largest fracture pits on the FJP-finished sample.

Because the fracture pits represent areas with increased contamination and potentially enhanced electric-field intensity, laser-damage testing was first conducted while targeting the seven largest fracture sites. Testing was conducted in the *R*-on-1 protocol using a starting fluence of 3 J/cm^2^. No damage to any of the fracture sites was observed at this fluence. The fluence was then ramped until laser-induced damage was observed. The damage-initiation fluences as they relate to pit size are shown in Fig. 6.

The lowest onset of damage occurred at a fluence of 4.1 J/cm^2^, while the smallest fracture pit was able to withstand laser-induced damage up to a fluence of 9.1 J/cm^2^. The data appears to show that below a pit size diameter of about 3500 μm^2^, the laser damage resistance is robust. Pits larger than this critical dimension demonstrate a reduced laser damage resistance where damage-initiation occurs between 4.1 and 6.4 J/cm^2^ regardless of their specific size. This size dependence may be due to either or both higher levels of trapped contamination and a greater effect on electric-field intensity. Laser-induced damage at these sites consisted of either ejected sections (fragments) or growth of the size of the fracture pit.

To further probe the effect of the FJP process on laser-induced damage behavior, we tested the FJP-finished surfaces while excluding those areas containing the largest fracture pits. Testing was conducted with the same laser source but instead employed the 1-on-1 protocol. Table 4 shows the 1-on-1 LIDT’s obtained for each of the surfaces. The LIDT values correspond to a damage probability of around 5% to 10%.

**Table 4:** The 1-on-1 LIDT’s of the four FJP-finished surfaces generated in this study.

Surface depth (μm)	LIDT (J/cm^2^)
0.16	7.9 ± 0.6
1.59	7.3 ± 0.5
4.19	8.6 ± 0.6
5.13	9.0 ± 0.7

**Figure 7:**
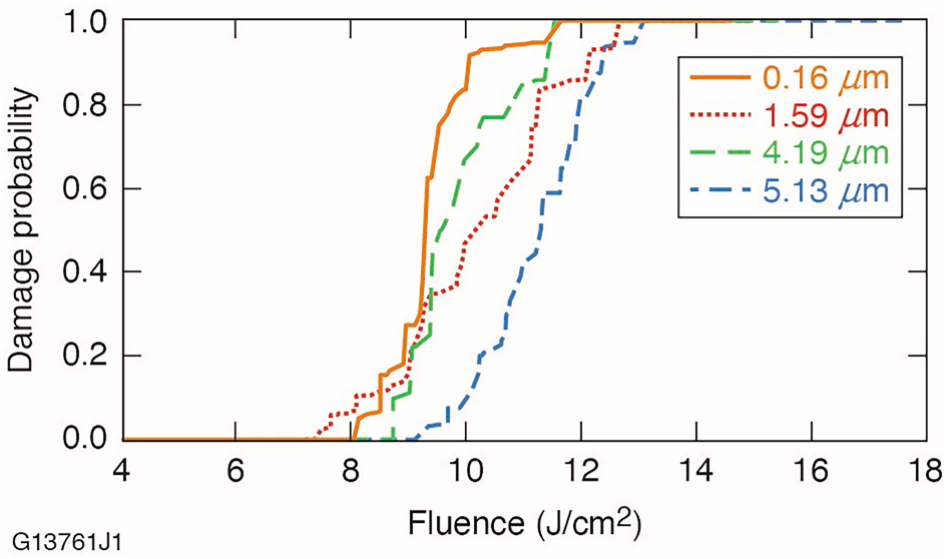
The 1-on-1 damage probability curves for the four FJP-finished surfaces.

The results in Table 4 indicate that for surface areas where fracture pits are not present, the laser damage resistance is markedly better. This finding is further exemplified when considering that the 1-on-1 protocol does not employ a series of low-fluence pulse ramps at the testing site. These fluence ramps used for *R*-on-1 testing are known to produce laser conditioning effects that can passivate or remove laser damage precursors^39^. The effect is particularly pronounced for KDP surfaces and can improve their *R*-on-1 LIDT by as much as 50% or more over the 1-on-1 value^40,41^. Although the 1-on-1 LIDT’s of each FJP-finished surface are statistically close in value, we gain important insight into their laser damage resistance when evaluating the damage probability curves shown in Fig. 7.

As demonstrated in Fig. 7, the laser damage resistance of the 5.13 μm surface exhibits the best performance. We believe this is primarily due to removal of a significant amount of pre-existing subsurface damage and contamination imparted from the grinding and pitch polishing processes. Since FJP can primarily remove material in the ductile mode, these subsurface damage features will be reduced substantially as the material removal depth increases. This behavior suggests that the LIDT of the FJP-finished material is related to the pre-FJP polishing conditions. Therefore, we postulate that material with improved initial polishing may yield a higher damage threshold after FJP.

## Conclusions

This article has demonstrated a freeform surface-figuring method by FJP, suitable for use with KDP optical crystals. Processing was enabled by using a nonaqueous slurry tailored to be most compatible with the physical properties of the material. As the material removal process progresses, fracture pits appear that are likely associated with dislocation defects in the near-surface of the bulk material. These pit defects harbor contamination from the slurry and represent areas of reduced laser damage resistance. We believe that improvements to the cleaning method should serve to remove contamination and improve the LIDT. Further study is ongoing to understand the root cause of these pits and to limit their formation during FJP. For surface areas outside of the fracture pits, the 1-on-1 laser damage behavior was robust and demonstrated a marked improvement at 5.13 μm of material removal. Taking into consideration the above results, we find that this method, even in its current state of development, will meet LIDT specifications for some laser systems such as OMEGA-60 (LLE) for implementation of a new type of freeform optic that supports polarization smoothing.

## Data Availability

Data underlying the results presented in this paper are not publicly available at this time but may be obtained from the authors upon reasonable request. Correspondence and requests for materials should be addressed to N.D.U.
